# A phase I and pharmacokinetic study of NK105, a paclitaxel-incorporating micellar nanoparticle formulation

**DOI:** 10.1038/sj.bjc.6603855

**Published:** 2007-06-26

**Authors:** T Hamaguchi, K Kato, H Yasui, C Morizane, M Ikeda, H Ueno, K Muro, Y Yamada, T Okusaka, K Shirao, Y Shimada, H Nakahama, Y Matsumura

**Affiliations:** 1 Department of Medicine National Cancer Center Hospital, 5-1-1 Tsukiji, Chuo-ku, Tokyo 104-0045, Japan; 2 Clinical Trial Coordinating Division, National Cancer Center Hospital, 5-1-1 Tsukiji, Chuo-ku, Tokyo 104-0045, Japan; 3 Investigative Treatment Division, Research Center for Innovative Oncology, National Cancer Center Hospital East, 6-5-1 Kashiwanoha, Kashiwa, 277-8577, Japan

**Keywords:** NK105, paclitaxel, polymer micelles, phase I study, DDS

## Abstract

This phase I study was designed to examine the maximum tolerated dose (MTD), the dose-limiting toxicities (DLTs), the recommended dose (RD) for phase II, and the pharmacokinetics of NK105, a new polymeric micelle carrier system for paclitaxel (PTX). NK105 was administered as a 1-h intravenous infusion every 3 weeks, without antiallergic premedication. The starting dose was 10 mg m^−2^, and the dose was escalated according to the accelerated titration method. Nineteen patients were recruited. The tumour types treated included pancreatic (*n*=11), bile duct (*n*=5), gastric (*n*=2), and colonic (*n*=1) cancers. Neutropenia was the most common haematological toxicity. A grade 3 fever developed in one patient given 180 mg m^−2^. No other grades 3 or 4 nonhaematological toxicities, including neuropathy, was observed during the entire study period. DLTs occurred in two patients given 180 mg m^−2^ (grade 4 neutropenia lasting for more than 5 days). Thus, this dose was designated as the MTD. Grade 2 hypersensitivity reactions developed in only one patient given 180 mg m^−2^. A partial response was observed in one patient with pancreatic cancer. The maximum concentration (*C*_max_) and area under the concentration (AUC) of NK105 were dose dependent. The plasma AUC of NK105 at 150 mg m^−2^ was approximately 15-fold higher than that of the conventional PTX formulation. NK105 was well tolerated, and the RD for the phase II study was determined to be 150 mg m^−2^ every 3 weeks. The results of this phase I study warrant further clinical evaluation.

Paclitaxel (PTX), an antimicrotubule agent, has a wide spectrum of antitumour activity including ovarian, breast, stomach, lung, and head and neck cancers (Rowinsky *et al*, 1990; Carney, 1996; Crown and O'Leary, 2000). The clinically used PTX preparation is a mixture of Cremophor EL and ethanol because of PTX's poor water solubility. However, the use of Cremophor EL is known to be associated with acute hypersensitivity reactions (Weiss *et al*, 1990; Rowinsky and Donehower, 1995; Kloover *et al*, 2004). Other PTX preparations that have been categorised as drug delivery systems (DDS) have also been developed. These preparations include Xyotax (polyglutamate-conjugated PTX; Singer *et al*, 2003; Boddy *et al*, 2005), Abraxane (PTX coated with albumin; Ibrahim *et al*, 2002; Deisai *et al*, 2003; Nyman *et al*, 2005), and Genexol-PM (a PTX micelle in which PTX has been simply solubilised; Kim *et al*, 2004). The common advantage shared by these formulations is that they are injectable intravenously without the mixture of Cremophor EL and ethanol. Among them, Abraxane has been approved for metastatic breast cancer by the Food and Drug Administration in the USA based on the results of a randomised phase 3 trial. In this trial, Abraxane demonstrated significantly higher response rates, compared with standard PTX, and a significantly longer time to progression (Gradishar *et al*, 2005). In addition, the incidence of grade 4 neutropenia was significantly lower for Abraxane than for PTX. However, peripheral sensory neuropathy was more common in the arm (Gradishar *et al*, 2005).

NK105 is a PTX-incorporating ‘core-shell-type’ polymeric micellar nanoparticle formulation (Hamaguchi *et al*, 2005). This particle can be injected intravenously without the use of Cremophor EL or ethanol as a vehicle. Therefore, NK105 is expected to possess a clinical advantage similar to that of the above-mentioned PTX formulations. The difference between NK105 and the other PTX dosage forms is that NK105 is expected to yield a markedly higher plasma and tumour area under the concentration (AUC), compared with those for the other PTX formulations. Moreover, regarding the toxic profiles, the repeated administration of NK105 to rats at 7-day intervals produced significantly fewer toxic effects on peripheral nerves than free PTX. Macromolecular drugs, including NK105, have been developed based on the characteristic macroscopic features of solid tumours, such as hypervasculature, the presence of vascular permeability factors stimulating extravasation within cancer, and the suppressed lymphatic clearance of macromolecules. These characteristics, which are unique to solid tumours, constitute the basis of the enhanced permeability and retention (EPR) effect (Matsumura and Maeda, 1986; Maeda *et al*, 2000; Duncan, 2003). The *in vivo* antitumour activity of NK105 was significantly more potent than that of free PTX, probably because of enhanced tumour exposure through the EPR effect (Hamaguchi *et al*, 2005).

We conducted a phase I clinical trial using NK105 in patients with advanced solid tumours. The objectives of this trial were to determine the maximum tolerated dose (MTD), the phase II recommended dose (RD), and the pharmacokinetics of NK105.

## PATIENTS AND METHODS

The protocol and all materials were approved by the Institutional Review Board of the National Cancer Center, Tokyo. This study was conducted in compliance with the Good Clinical Practice Guidelines of the International Conference on Harmonization and the Declaration of Helsinki Principles. Written informed consent was obtained from all the patients.

### Therapeutic agent

NK105 was supplied by Nippon Kayaku Co. Ltd. (Tokyo, Japan) in 20-ml glass vials containing a dose equivalent to 30 mg of PTX. When reconstituted in 10 ml of 5% glucose solution and diluted with a total volume of 250 ml of 5% glucose, the reconstituted solution was stable for 24 h at room temperature. In our preclinical study, DLS and HPLC analysis showed that less than 2% of PTX incorporated in the micelles was released for 24 h at room temperature (data not shown).

**Figure 1:**
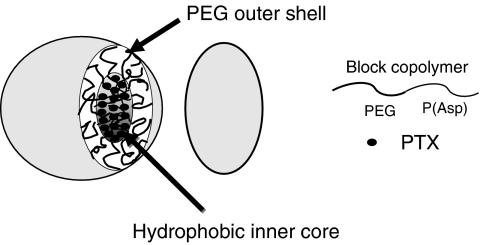
Schematic structure of NK105. A polymeric micelle carrier of NK105 consists of a block copolymer of PEG (molecular weight of about 12 000) and modified polyaspartate. PEG is believed to be the outer shell of the micelle. PEG is believed to form the outer shell of the micelle. NK105 has a highly hydrophobic inner core, and therefore can entrap a sufficient amount of PTX.

Figure 1 shows the schematic structure of NK105, a PTX-entrapped polymeric micelle formulation. The NK105 polymers were constructed using polyethylene glycol (PEG) as the hydrophilic component and modified polyaspartate as the hydrophobic component. PEG is believed to form the outer shell of the micelle, producing a ‘stealth’ effect that enables NK105 to avoid being captured by the reticuloendothelial system.

The modified polyaspartate chain is hydrophobic and is believed to form the hydrophobic inner core of the micelles in aqueous media. The hydrophobic inner core enables NK105 to entrap a sufficient amount of PTX. NK105 has a diameter of about 90 nm (Hamaguchi *et al*, 2005).

### Patients

Patients with solid tumours refractory to conventional chemotherapy and for whom no effective therapy was available were eligible for enrolment in this study, provided that the following criteria were met: a histologically confirmed malignant tumour; a performance status of ⩽2; an age of ⩾20 and <75 years; a normal haematological profile (neutrophil count ⩾2000 mm^−3^, platelet count ⩾100 000 mm^−3^, hemoglobin ⩾9 g dl^−1^); normal hepatic function (total bilirubin level ⩽1.5 mg dl^−1^, AST and ALT ⩽2.5 times the upper normal limit); normal renal function (serum creatinine ⩽1.5 mg dl^−1^); normal cardiac function (New York Heart Association (NYHA) classification of ⩽1); normal pulmonary function (PaO_2_⩾60 mm Hg); no chemotherapy within 4 weeks (6 weeks for nitrosourea or mitomycin C) of the administration of NK105; and a life expectancy of more than 2 months. Patients with serious infections (including hepatitis B, hepatitis C, or HIV) were ineligible for enrolment in the study. Patients who had been previously treated with a taxane were excluded because of assessing neuropathy. Patients were also excluded if they were pregnant or lactating. Additionally, any patient whom the investigators considered ineligible was excluded.

### Drug administration

NK105 was dissolved in 5% glucose solution for injection at room temperature. NK105 was administered intravenously without in-line filtration and without premedication. NK105 solution was infused using an electric pump at a speed of 250 ml h^−1^.

### Dosage and dose escalation

The starting dosage of NK105 was 10 mg m^−2^, which is one-third of the toxic dose low in dogs. NK105 was administered once every 3 weeks, and the treatment was continued unless a severe adverse event or disease progression was observed. Dose escalation was performed according to the previously described accelerated titration method (Simon *et al*, 1997; Matsumura *et al*, 2004).

Toxicity was graded from 1 to 4 using the National Cancer Institute Common Toxicity Criteria (version 2.0). Intrapatient dose escalation was not permitted. The MTD was defined as the level at which two out of six patients experienced dose-limiting toxicities (DLTs). The recommended dosage for a phase II trial was defined by the Efficacy and Safety Assessment Committee based on the safety, pharmacokinetics, and efficacy results of this trial. DLT was defined as grade 4 neutropenia lasting more than 5 days, a platelet count of less than 25 000 *μ*l^−1^, or grade 3 or higher non-haematological toxicity, with the exception of nausea, vomiting, appetite loss, and hypersensitivity.

### Pretreatment assessment and follow-up care

A complete medical history and physical examination, performance status evaluation, complete blood cell count (CBC), blood chemistry, urinalysis, electrocardiogram (ECG), and a computed tomography (CT) examination were performed in each patient. Other examinations were performed only in the presence of a specific clinical indication. Patients were physically examined every day until the second administration of NK105; CBC and blood chemistry tests were performed on day 3 and weekly thereafter. An ECG examination was repeated before each administration of NK105. Tumour marker levels were also measured before every administration. Tumour response was evaluated according to the Response Evaluation Criteria in Solid Tumors criteria (Therasse *et al*, 2000).

### Liquid chromatography/tandem mass spectrometry determination of PTX concentrations

The PTX concentrations determined in the present phase I study represented the total drug concentrations (both micelle-entrapped and released). It was difficult to measure released PTX and micelle-entrapped PTX separately, because the equilibrium between both forms could not keep constant during the separating procedure. PTX was extracted from human plasma (0.2 ml) or urine (0.5 ml) by deproteinisation with acetonitrile. The quantifications of PTX in plasma and urine were performed using liquid chromatography/tandem mass spectrometry. Reversed-phase column-switching chromatography was conducted using an ODS column and detection was enabled by electrospray ionisation of positive mode.

### Pharmacokinetic analysis

The following pharmacokinetic parameters were calculated for each patient using a non-compartmental model using the WinNonlin Professional version 4.1 program (Pharsight Corporation, Mountain View, CA, USA). The maximum concentration (*C*_max_) was the maximum observed plasma concentration of PTX, and the time-to-the-maximum concentration (*T*_max_) was the time corresponding to *C*_max_. The area under the concentration (AUC)–time curve from time zero up to the last quantifiable time point (AUC_0–*t*_) was calculated using the linear trapezoidal rule, and the area under the concentration–time curve from zero until infinity (AUC_0–inf._) was calculated as the sum of AUC_0–*t*_ and the extrapolated area under the zero moment curve from the last quantifiable time point to infinity calculated by dividing the plasma concentration of the last quantifiable time point (observed value) by the elimination rate constant. The half-life of the terminal phase (*t*_1/2*Z*_) was calculated as log_e_ 2/*λz*, where *λz* is the elimination rate constant calculated from the terminal linear portion of the log of the concentration in plasma. Total clearance (CL_tot_), the volume of distribution at steady state (*V*_ss_), and renal clearance (CL_r_) were calculated using the following equations, where *D* is the dose and AUMC_inf._ the area under the first moment curve from time zero until infinity: 
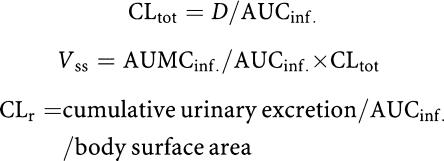


## RESULTS

### Patient characteristics

Nineteen eligible patients were recruited for the study (Table 1). All the patients had received chemotherapy before enrolment. Prior therapies ranged from 1 to 3 regimens of chemotherapy. None of the patients had received taxane chemotherapy. All the patients were included in the safety and response analyses.

### Dosing

Dosage escalation started at 10 mg m^−2^ and was increased up to 180 mg m^−2^. In total, 73 administrations were performed in 19 patients. Eighteen patients received more than two administrations. The maximum number of treatments was 14 courses at 150 mg m^−2^; the average number of administrations at all levels was 3.8 courses. Up until 80 mg m^−2^, grade 2 toxicity was not observed during the first course.

According to the original protocol, the dosage of NK105 should have been doubled for each escalation until grade 2 toxicity. However, the safety committee recommended that the dosage should be raised by 40% instead of 100% at 110 mg m^−2^ and that a modified Fibonacci escalation method should be implemented. Therefore, we recruited three patients at dosage level 5 (110 mg m^−2^) and re-started the dose identification study using a modified Fibonacci method.

### Haematological toxicity

Significant myelosuppression was not observed up to level 4 (80 mg m^−2^). At level 7 (180 mg m^−2^), two out of five patients appeared to have acquired DLTs, namely grade 4 neutropenia lasting for more than 5 days. On the basis of these results, 180 mg m^−2^ was considered to be the MTD, with neutropenia as the DLT. Since a dosage of 150 mg m^−2^ was considered to be the recommended dosage for phase II studies, an additional four patients were enrolled at a dosage of 150 mg m^−2^; one patient developed DLT, namely grade 4 neutropenia lasting for more than 5 days (Table 2). During the entire period of this study, G-CSF was never used to rescue patients.

### Nonhaematological toxicity

The NK105 injection was generally uneventful and well tolerated in terms of nonhaematological toxicities (Table 2). Most of the toxicities were grade 1; none of the patients manifested grade 4 toxicity. A few patients developed a grade 1 elevation in AST or ALT, but these changes were transient. Pain or local toxicity in the area of the injection was not observed in any of the patients treated with NK105. No infusion-related reactions were observed; such reactions sometimes occur during liposomal drug administration. Patients were not premedicated with steroids or antihistamines. Only one patient at 180 mg m^−2^ developed grade 2 hypersensitivity. After the first course, the patient received premedication of hydrocortisone and did not develop such hypersensitivity after that. The other 18 patients did not experience any hypersensitivity during the study. Neuropathy occurred in a typical stocking/glove distribution and was manifested by numbness. Three patients at level 6 (150 mg m^−2^) and three patients at level 7 (180 mg m^−2^) experienced grade 1 neurotoxicity during 1 cycle. Of the four patients who received multicycle treatment more than five times, only three patients developed grade 2 neuropathy and the other patient developed grade 1 neuropathy. Even one patient who received 14 cycles of treatment experienced only grade 2 neuropathy.

### Pharmacokinetics

**Figure 2:**
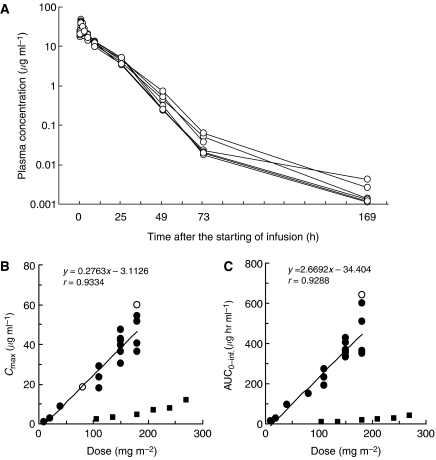
(**A**) Individual plasma concentrations of PTX in seven patients following 1-h intravenous infusion of NK105 at a dose of 150 mg m^−2^. (**B**) Relationships between dose and *C*_max_, and (**C**) between dose and AUC_0−inf._ of PTX in patients following 1-h intravenous infusion of NK105. Regression analysis for dose *vs C*_max_ was applied using all points except one patient at 80 mg m^−2^ whose medication time became 11 min longer and one patient at 180 mg m^−2^ who had medication discontinuation and steroid medication. (Plots were shown as open circle). Regression analysis for dose *vs* AUC_0−inf._ was applied using all points except one patient who had medication discontinuation and steroid medication. (Plot was shown as open circle.) Relationships between dose and *C*_max_, and AUC_0−inf._ in patients following conventional PTX administration were plotted (closed square, see Tamura *et al*, 1995).

The plasma concentrations of PTX after the intravenous infusion of NK105 were determined in each of the patients enrolled at a dose of 150 mg m^−2^ (Figure 2A). The *C*_max_ (Figure 2B) and AUC (Figure 2C) increased as the doses were escalated from 10 to 180 mg m^−2^. The pharmacokinetic parameters are summarised in Table 3. The *t*_1/2*Z*_ ranged from 7.0 to 13.2 h, and a slight tendency towards a dose-dependent extension of this parameter was observed. The CL_tot_ ranged from 280.9 to 880.4 ml h^−1^ m^−2^, and the *V*_ss_ ranged from 3668.9 to 10 400.3 ml m^−2^. Although these parameters were slightly reduced depending on the dose, linear pharmacokinetics was assumed to have been observed in the dose range from 10 to 180 mg m^−2^. The AUC of NK105 at 150 mg m^−2^ (recommended phase II dose) was about 15-fold larger than that of conventional PTX at dose of 210 mg m^−2^ (conventional dose for a 3-week regimen in Japanese patients) (Tamura *et al*, 1995). The *V*_ss_ and CL_tot_ of NK105 were significantly lower than those of conventional PTX.

The cumulative urinary excretion rates of PTX (0–73 h) after the administration of NK105 were 2.8–9.2%. These values were low, similar to those reported after the administration of conventional PTX (Tamura *et al*, 1995). The CL_r_ ranged from 11.7 to 66.4 ml h^−1^ m^−3^, and was slightly decreased with the dose. Since the ratio of CL_r_ to CL_tot_ was 3–9%, CL_r_ hardly contributed to CL_tot_.

### Therapeutic response

**Figure 3:**
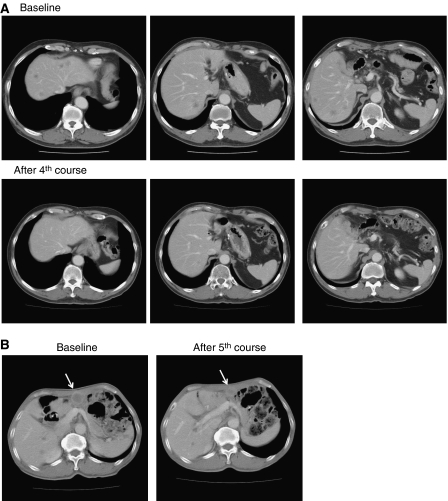
Serial CT scans. (**A**) A 60-year-old male with pancreatic cancer who was treated with NK105 at a dose level of 150 mg m^−2^. Baseline scan (upper panels) showing multiple metastasis in the liver. Partial response, characterized by a more than 90% decrease in the size of the liver metastasis (lower panels) compared with the baseline scan. The antitumour response was maintained for nearly 1 year. (**B**) A 64-year-old male with stomach cancer who was treated with NK105 at a dose level of 150 mg m^−2^. Baseline scan (left panel) showing a peritoneal metastasis and liver metastasis. About 40% reduction (right panel) was observed in peritoneal metastasis, but not in the liver metastasis after fifth course.

Six patients (two gastric, two bile duct, one colon, and one pancreatic) were evaluated as having had a stable disease for longer than 4 weeks at the time of the study's completion. A partial response was seen in a patient with metastatic pancreatic cancer who had been treated at 150 mg m^−2^, and in whom the size of the liver metastasis had decreased by more than 90%, compared to the baseline scan (Figure 3A). This patient had previously undergone treatment with gemcitabine. The antitumour response was maintained for nearly 1 year. In a patient with stomach cancer who was treated at 150 mg m^−2^, about 40% reduction was observed in a peritoneal metastasis, but a liver metastasis remained stable (Figure 3B).

## DISCUSSION

The observed toxicities of NK105 were similar to those expected for conventional PTX. The DLT was neutropenia. The recommended phase II dose using a 3-week schedule was determined to be 150 mg m^−2^. This recommended dose of NK105 is less than that of conventional PTX (210 mg m^−2^). Since the plasma AUC of the recommended dose of NK105 was 15- to 20-fold higher than that of the recommended dose of conventional PTX (210 mg m^−2^), whether the so-called therapeutic window of NK105 is wider than that of conventional PTX should be determined in a future phases II or III trial, although the therapeutic window of NK105 appears to be wider than that of free PTX in mice experiments (Hamaguchi *et al*, 2005).

In general, haematological toxicity was mild and well managed in this trial. PTX is known to cause cumulative peripheral neuropathy resulting in the discontinuation of treatment with PTX. At a dose of 150 mg m^−2^, three out of seven patients experienced only grade 1 neuropathy during the first cycle. Since the patients enrolled in this trial had almost intractable cancer, such as pancreatic or stomach, a relatively small number of patients received multiple cycles of treatment. Therefore, NK105-related neurotoxicity could not be evaluated in this study. However, three out of four patients who received more than five cycles of treatment experienced transient grade 2 peripheral neuropathy, and other patient developed transient grade 1 peripheral neuropathy. Future phase II trials may clarify whether NK105 is less toxic in terms of peripheral neuropathy when compared with conventional PTX, Abraxane, and other PTX compounds. Another characteristic adverse effect of PTX is hypersensitivity, which may be mainly caused by Cremophor EL. Since NK105 is not formulated in a Cremophor EL-containing solvent, we presumed that hypersensitivity would be diminished. Indeed, the results of this clinical trial show that NK105 can be administered safely as a short infusion (1 h) without the administration of antiallergic agents like dexamethasone and antihistamine, although one patient at 180 mg m^−2^ developed transient grade 2 hypersensitivity at the first course. Therefore, NK105 may offer advantages in terms of safety and patient convenience and comfort.

The pharmacokinetic analysis of NK105 suggests that the distribution of PTX-incorporating micelles is mostly restricted to the plasma and, in part, to extracellular fluids in the body. This is consistent with data obtained in a preclinical study (Hamaguchi *et al*, 2005) showing that the distribution of NK105 in tissues is characterised by an EPR effect, similar to that of tumour and inflammatory lesions, or by the presence of a reticuloendothelial system. When compared with conventional PTX at a dose of 210 mg m^−2^ (conventional dose for a 3-week regimen in Japanese patients), NK105 at a dose of 150 mg m^−2^ (recommended phase II dose) exhibited more than 15-fold larger plasma AUC and a 26-fold lower CL_tot_. The larger plasma AUC is consistent with the stability of the micelle formulation in plasma. The *V*_ss_ of NK105 was 13-fold lower than that of conventional PTX. This suggests that PTX may have a relatively lower distribution in normal tissue, including normal neural tissue, following NK105 administration. Regarding the drug distribution in tumours, nanoparticle drug carriers have been known to preferentially accumulate in tumour tissues utilising the EPR effect (Matsumura and Maeda, 1986; Maeda *et al*, 2000; Duncan, 2003). We speculate that NK105 accumulates more in tumour tissues than free PTX, since NK105 is very stable in the circulation and exhibits a markedly higher plasma AUC than free PTX. Moreover, a polymeric micelle carrier system for a drug has the potential to enable the sustained release of the drug inside a tumour following the accumulation of micelles in the tumour tissue (Hamaguchi *et al*, 2005; Uchino *et al*, 2005; Koizumi *et al*, 2006). Regarding NK105 in particular, this sustained release may begin at a PTX-equivalent dose of <1 *μ*g ml^−1^ (data not shown). Consequently, the released PTX is distributed throughout the tumour tissue where it kills the cancer cells directly.

In the present study, NK105 appeared to exhibit characteristic pharmacokinetics different from those of other PTX formulations including conventional PTX, Abraxane, Genexol-PM, and Xyotax. For example, previous clinical PK data at each phase II recommended dose shown that plasma AUC and *C*_max_ were 11.58 and 3.1 in Genexol-PM (Table 4). The antitumour activities seen in two patients with intractable cancers are encouraging. In addition, we recently demonstrated in preclinical study that combined NK105 chemotherapy with radiation exerts a significantly more potent antitumour activity, compared with combined PTX therapy and radiation (Negishi *et al*, 2006). This data on NK105 justifies its continued clinical evaluation.

## Figures and Tables

**Table 1:** Patient characteristics

Number of patients	19
Male/female	13/6
	
*Age (years)*	
Median	57
Range	43–72
	
*ECOG PS*	
Median	0
0	10
1	9
	
*Prior treatment*	
Chemotherapy regimens	
Median	1
Range	1–3

**Table 2:** Haematological and nonhaematological toxicities (cycle 1 and all cycles)

	**10–110 mg m^−2^ (*n*=7) grade**				**150 mg m^−2^ (*n*=7) grade**				**180 mg m^−2^ (*n*=7) grade**			
	**1**	**2**	**3**	**4**	**1**	**2**	**3**	**4**	**1**	**2**	**3**	**4**
*Cycle 1*												
Leukopenia	2	0	2	0	1	5	1	0	1	1	3	0
Neutropenia	1	0	1	1	0	2	1	3^a^	0	0	3	2^b^
Thrombocytopenia	1	0	0	0	2	0	0	0	4	0	0	0
Hemoglobin	1	0	0	0	2	2	0	0	1	0	0	0
Neuropathy	0	0	0	0	3	0	0	0	3	0	0	0
Myalgia	1	0	0	0	3	0	0	0	2	1	0	0
Arthralgia	1	0	0	0	4	0	0	0	3	0	0	0
Hypersensitivity	0	0	0	0	0	0	0	0	0	1	0	0
Rash	1	0	0	0	1	3	0	0	4	0	0	0
Fatigue	1	0	0	0	5	0	0	0	4	0	0	0
Fever	2	0	0	0	2	0	0	0	1	0	1	0
Anorexia	0	0	0	0	3	0	0	0	1	0	0	0
Nausea	1	0	0	0	1	0	0	0	1	0	0	0
Stomatitis	0	0	0	0	1	0	0	0	1	0	0	0
Alopecia	3	0	—	—	5	0	—	—	5	0	—	—
												
*All cycles*												
Leukopenia	3	0	2	0	1	4	2	0	1	1	3	0
Neutropenia	1	0	1	1	1	1	1	4	0	0	3	2
Thrombocytopenia	1	0	0	0	3	0	0	0	4	0	0	0
Hemoglobin	1	0	0	0	1	5	0	0	1	0	0	0
Neuropathy	2	0	0	0	1	3	0	0	4	0	0	0
Myalgia	1	1	0	0	3	0	0	0	2	1	0	0
Arthralgia	2	0	0	0	4	0	0	0	3	0	0	0
Hypersensitivity	0	0	0	0	0	0	0	0	0	1	0	0
Rash	1	0	0	0	3	3	0	0	4	0	0	0
Fatigue	3	0	0	0	5	1	0	0	4	0	0	0
Fever	3	0	0	0	3	1	0	0	1	0	1	0
Anorexia	2	1	0	0	2	1	0	0	2	0	0	0
Nausea	1	0	0	0	1	0	0	0	2	0	0	0
Stomatitis	1	0	0	0	2	0	0	0	1	0	0	0
Alopecia	2	2	—	—	4	3	—	—	4	1	—	—

a One of three patients developed DLT, namely grade 4 neutropenia lasting for more than 5 days.

b These two patients developed DLT, namely grade 4 neutropenia lasting for more than 5 days.

**Table 3:** Pharmacokinetic parameters

	**Dose (mg m^−2^)**	***n***	***C*_max_** **(*μ*g ml^−1^)**	**AUC_0−inf._ (*μ*g h ml^−1^)**	***t*_1/2_ (h)**	**CL_tot_ (ml h^−1^ m^−2^)**	***V*_ss_ (ml m^−2^)**	**UE**^a^ **(%)**	**CL_r_ (ml h m^−2^)**
NK105	10	1	0.9797	11.4	9	880.4	10 400.3	7.5	66.4
	20	1	2.8971	29.1	8.5	687.9	8027	8.6	59.4
	40	1	8.8334	93.9	13.2	426.1	5389.8	5.2	22
	80	1	18.4533	149.3	7	535.8	5875.8	4.7	25.3
	110	3	23.3924	232	9.7	483.3	5881.2	7.6	35.6
			±5.6325	±39.1	±1.6	±82.7	±1512.0	±1.7	±6.9
	150	7	40.1699	369.8	10.6	408.6	4527.1	5.3	21.6
			±5.5334	±35.2	±1.3	±37.3	±639.5	±1.5	±6.5
	180	4^b^	45.6278	454.5	11.3	416.5	4983.4	5.9	23.7
			±8.6430	±119.1	±0.6	±104.7	±887.5	±1.4	±4.2

a UE, urinary excretion.

b One patient at 180 mg m^−2^ level was omitted from the calculation of summary pharmacokinetic parameters, as there was administrating interruption for developing allergic reactions.

**Table 4:** Pharmacokinetic parameters

	**Dose (mg m^−2^)**	***n***	***C*_max_ (*μ*g ml^−1^)**	**AUC_0−inf._ (*μ*g h^−1^ ml^−1^)**	***t*_1/2_ (h)**	**CL_tot_ (ml h^−1^ m^−2^)**	***V*_ss_ (ml m^−2^)**	**UE (%)**	**CL_r_ (ml h m^−2^)**
NK105	150	7	40.1699	369.8	10.6	408.6	4527.1	5.3	21.6
			±5.5334	±35.2	±1.3	±37.3	±639.5	±1.5	±6.5
PTX	210	5	6.744	23.18	13.3	10740	58 900	9.45	1020
			±2.733	±10.66	±1.5	±4860	±24 700	±3.76	±648
XYOTAX^a^	233	4	NA	1583	120	276	6200	NA	NA
				±572	±28	±63	±2100		
Abraxane	300	5	13.52	17.61	14.6	17 700	370 000	NA	NA
			±0.95	±3.70	±2.04	±3894	±85 100		
Genoxol-PM	300	3	3.107	11.58	11.4	29 300	NA	NA	NA
			±1.476	±4.28	±2.4	±13 800			

a Conjugated taxanes.
